# Multimodal single cell analysis infers widespread enhancer co-activity in a lymphoblastoid cell line

**DOI:** 10.1038/s42003-023-04954-4

**Published:** 2023-05-26

**Authors:** Chaymae Ziyani, Olivier Delaneau, Diogo M. Ribeiro

**Affiliations:** 1 Department of Computational Biology, University of Lausanne, Lausanne, Switzerland; 2 Swiss Institute of Bioinformatics (SIB), Lausanne, Switzerland

**Keywords:** Gene regulation, Gene regulatory networks, Transcriptomics

## Abstract

Non-coding regulatory elements such as enhancers are key in controlling the cell-type specificity and spatio-temporal expression of genes. To drive stable and precise gene transcription robust to genetic variation and environmental stress, genes are often targeted by multiple enhancers with redundant action. However, it is unknown whether enhancers targeting the same gene display simultaneous activity or whether some enhancer combinations are more often co-active than others. Here, we take advantage of recent developments in single cell technology that permit assessing chromatin status (scATAC-seq) and gene expression (scRNA-seq) in the same single cells to correlate gene expression to the activity of multiple enhancers. Measuring activity patterns across 24,844 human lymphoblastoid single cells, we find that the majority of enhancers associated with the same gene display significant correlation in their chromatin profiles. For 6944 expressed genes associated with enhancers, we predict 89,885 significant enhancer-enhancer associations between nearby enhancers. We find that associated enhancers share similar transcription factor binding profiles and that gene essentiality is linked with higher enhancer co-activity. We provide a set of predicted enhancer-enhancer associations based on correlation derived from a single cell line, which can be further investigated for functional relevance.

## Abstract (web summary)

Analysis of scRNA-seq and scATAC-seq data from single lymphoblastoid cells generates a set of predicted enhancer-enhancer associations, where co-active enhancers are more likely to be bound by similar transcription factors.

## Introduction

Gene expression regulation is an essential biological process across all organisms and allows for different genes to be activated in a cell type-specific manner, leading to distinct morphologies and cellular functions^1,2^. Gene expression is controlled by genomic regulatory elements such as promoters, insulators, and enhancers^3^. Dysregulation of these elements can lead to a variety of illnesses such as cancer, metabolic syndromes and developmental disorders^4,5^. By harbouring transcription factor binding sites (TFBS), enhancers regulate the spatio-temporal patterns and expression levels of nearby genes irrespective of the position, distance, or orientation relative to the target promoter^6^. To achieve robust expression as well as tight control across cellular contexts, genes utilise multiple enhancers, often with redundant action^7–10^. Indeed, intricate networks of gene expression and regulatory element activity have been revealed in multiple human cell lines^11–13^. In particular, shadow enhancers – sets of enhancers that regulate the same gene, with overlapping activity patterns in space and time – are remarkably abundant and key in controlling developmental gene expression^10,14–16^. Indeed, the action of shadow enhancers has been shown to confer phenotypic robustness to loss-of-function mutations in individual enhancers in loci linked to limb development^10^.

Recent studies have identified enhancers and gene-enhancer links across most human tissues and cell types from ATAC-seq data, ChIP-seq, RNA-seq and CRISPR perturbations^17–19^. However, these studies do not provide information regarding the dynamics of enhancer activity during gene expression regulation and multiple open questions remain, such as whether enhancers targeting the same gene display simultaneous activity, and whether some combinations of enhancers are more often co-active than others. In particular, only a few studies have focused on studying enhancer-enhancer associations in the context of gene regulation^20,21^. The development of multimodal single cell datasets, particularly those assessing chromatin status (e.g. scATAC-seq) and gene expression (scRNA-seq) in the same single cells^22–24^ allow us to directly couple both activity profiles and address these questions at a large-scale.

Here, we exploit the SHARE-seq dataset^22^ with scRNA-seq and scATAC-seq across 24,844 cells in a single human cell line (lymphoblastoid cell line, LCL) to measure enhancer co-activity during gene expression. Starting from cis gene-enhancer associations that we previously identified^25^, for each gene, we correlated the activity levels of all their nearby (within 1 Mb) associated enhancers. Across 6944 expressed genes associated with enhancers, we identified 89,885 enhancer-enhancer associations, amounting to 70.8% of all possible enhancer pairs. Our results suggest the pervasiveness of enhancers with shadow enhancer potential and highlight some of their features such as (i) higher sharing of transcription factor binding sites and (ii) higher enhancer co-activity in essential genes. Our predicted enhancer-enhancer associations help pave the way for further studies of their functional relevance and role in gene regulation. Knowledge of the relevant regulatory element circuitry, such as which enhancers or combinations of enhancers are relevant for the expression of genes, would allow us to better predict the effect of the hundreds of thousands of genome-wide association studies (GWAS) hits falling in non-coding regions.

## Results

### Enhancer-enhancer association predictions from multimodal single cell data

**Fig. 1:**
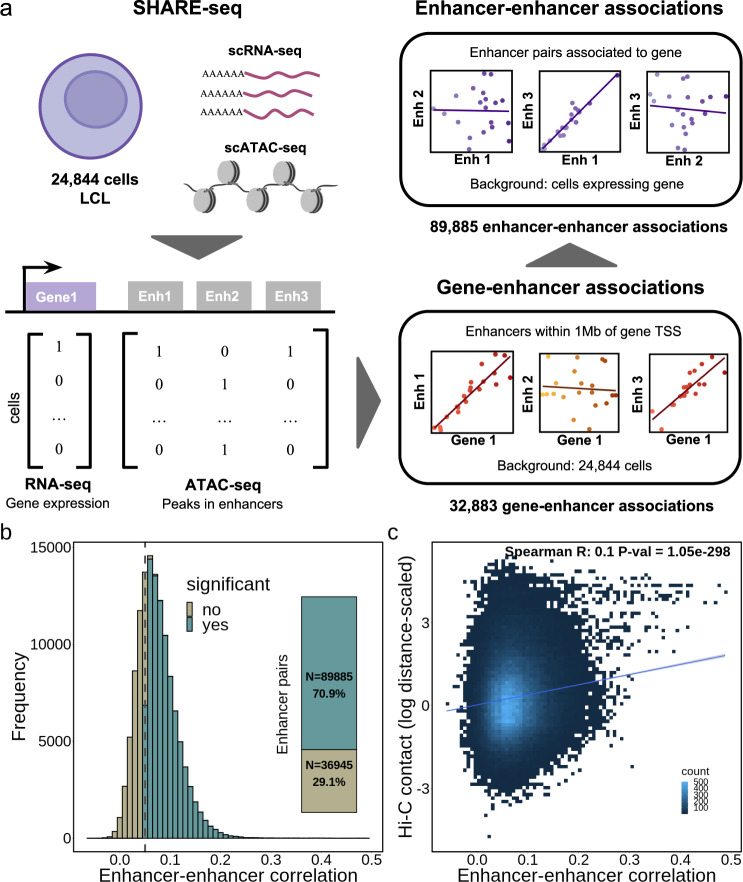
Enhancer-enhancer co-activity overview. **a** Scheme of the approach used to determine gene-enhancer associations and enhancer-enhancer associations from SHARE-seq data. Enhancer-enhancer associations were calculated for pairs of enhancers significantly associated with genes; **b** Enhancer-enhancer association correlation distribution (*N* = 126,830). The inner plot denotes the percentage of significant associations (green colour, FDR < 5% and absolute correlation > 0.05); **c** Hi-C contacts (log distance-scaled, 5 kb resolution) per enhancer-enhancer correlation value (*N* = 126,830).

We explore enhancer regulation in a gene-centric way. We have previously exploited the multimodal SHARE-seq single cell dataset^22^, to identify 32,883 gene-enhancer pairs (6944 distinct genes, 7551 distinct enhancers) with correlated activity^25^. Briefly, these gene-enhancer associations were identified using 24,844 LCL cells which contained both scRNA-seq and scATAC-seq data. To focus on enhancer regions, only scATAC-seq peaks overlapping LCL-specific enhancer regions from the EpiMap repository^19^ were considered (“active enhancers” and “genic enhancers” from GM12878, see Methods). We then correlated gene expression and the activity of nearby enhancers (±1 Mb around gene TSS) across cells to identify significant associations (FDR < 5% and absolute Pearson correlation > 0.05, Fig. 1a). Out of 16,463 protein-coding genes tested, 6944 were associated with at least one enhancer and 5087 genes were associated with two or more enhancers (max = 35 enhancers, mean = 4.7), which we further analyse for enhancer-enhancer associations.

While several studies identified gene-enhancer associations^17,19,22^, the association between multiple enhancers has not yet been explored at a large-scale. Here, using the same LCL SHARE-seq dataset^22^, we explore the co-activity between enhancers associated with a certain gene. For this, we measure the correlation of enhancer activity (based on scATAC-seq peaks overlapping EpiMap enhancers), for the 5087 genes previously associated with more than one enhancer^25^. Briefly, for each gene we (i) define the set of cells (with RNA-seq and ATAC-seq data) in which the gene is expressed (defined as non-zero expression, mean = 2938 cells per gene), (ii) we gather all enhancer regions associated with the gene (within 1 Mb of the gene TSS, mean = 6.1 enhancers per gene), and finally, (iii) for each pair of enhancers, we measure the correlation between their activity (0 or 1) across the cells expressing the gene (see Methods, Fig. 1a). Using this approach, we performed 126,830 correlation tests, of which 89,885 (70.9%) were deemed significant (FDR < 5%, absolute correlation > 0.05, Supplementary Data 1). The significant associations comprised 4822 distinct genes and 6743 enhancers and all were positively correlated (Fig. 1b). Different significance cutoffs were explored, with 22.1% (FDR 5% and absolute correlation coefficient > 0.1) to 88.6% (FDR 5%, no correlation cutoff) of the tests being deemed significant (Supplementary Fig. 1a). We observed similar proportions of significant enhancer-enhancer associations across cutoffs when considering ABC enhancers and gene-enhancer associations (e.g. 62.9% significant enhancer-enhancer associations with FDR < 5% and correlation >0.05, Supplementary Fig. 1b). We opted for an absolute correlation coefficient cutoff of 0.05 (and FDR 5%) as a moderately strict cutoff, with 70.9% of the tests deemed significant for the SHARE-seq dataset. As a comparison, only 18.4% of the 2,878,013 enhancer-enhancer association tests performed when considering all enhancers within 1 Mb of the gene TSS (instead of only the enhancers associated with the same gene) were found significant with the same cutoff (Supplementary Data 2). In fact, only 13.3% of the enhancer pairs were found associated when considering pairs of enhancers not associated with the gene (Supplementary Fig. 2). This indicates that if several enhancers are associated with the same gene, they are more likely to be significantly associated between themselves, as expected.

Next, we assessed whether enhancer-enhancer associations are kept when adjusting for the expression of associated genes. For this, we performed partial correlation for all 126,830 gene-enhancer-enhancer combinations previously tested across all 24,844 SHARE-seq cells (see Methods, Supplementary Data 3). We observed high concordance between correlation coefficients (Spearman R = 0.83, *p*-value < 2.2e^−308^, Supplementary Fig. 3a), although correlation coefficients are generally lower with partial correlation. Correlation p-values are also consistent (Spearman R = 0.71, *p*-value < 2.2e^−308^, Supplementary Fig. 3b), with only 35 enhancer pairs out of the 89,885 significantly associated enhancer pairs with a partial correlation FDR above 5%. Indeed, partial correlation is clearly higher in the 89,885 significant enhancer pairs compared to non-significant pairs (Wilcoxon test *p*-value < 2.2e^−308^, Supplementary Figure 3c). For instance, 49.7% of the significant enhancer pairs have partial correlation >0.05, compared to only 2.1% of non-significant enhancer pairs (Fisher’s exact test odds ratio = 24.02, *p*-value < 2.2e^−308^, Supplementary Fig. 3d). This shows that the predicted enhancer-enhancer associations display significant correlation when accounting for gene expression.

To support our predicted enhancer-enhancer associations, we analysed publicly available Hi-C data (5 kb resolution) for LCLs^26^. We found that the correlation level of the 126,830 enhancer-enhancer association tests correlates with Hi-C contact intensities (Spearman R = 0.1, *p*-value < 1.1e^−298^, Fig. 1c). Moreover, Hi-C contacts between enhancer-enhancer pairs were higher than in distance-matched control regions (Wilcoxon test *p*-value < 2.2e^−308^, see Methods, Supplementary Fig. 4a). Indeed, 88,283 (69.6%) out of the 126,830 enhancer-enhancer pairs displayed higher Hi-C contacts than expected by their distance (Supplementary Fig. 4b). In addition, when considering a biological replicate with 2788 SHARE-seq cells with both scRNA-seq and scATAC-seq (instead of the 24,844 cells used for discovery), we found significant concordance between the enhancer-enhancer correlation levels of the replicates (Spearman R = 0.22, *p*-value < 2.2e^−308^, Supplementary Fig. 5). Likewise, we observed similar enhancer co-activity patterns and Hi-C contact correlations when considering an alternative public multimodal dataset comprising 13,311 peripheral blood mononuclear cells (PBMCs, see Methods, Supplementary Fig. 6). Together, these results support the use of multimodal single cell ATAC-seq and RNA-seq data to predict enhancer co-activity associations involved in regulating the same gene.

### Prevalence of enhancer co-activity across genes

**Fig. 2:**
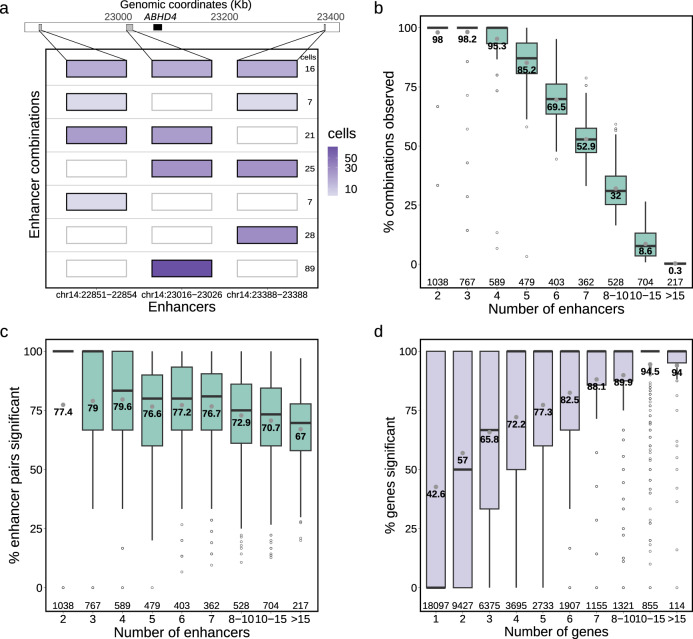
Frequency of co-active enhancers. **a** Enhancer combinations observed in single cells for the *ABHD4* example gene with three associated enhancers. All seven possible combinations between three enhancers are represented (y-axis), with colour intensity mapped to the number of cells in which the combinations are observed; **b** Percentage of enhancer combinations observed in at least one cell (y-axis) per number of enhancers significantly associated with the gene (x-axis). Grey dots and nearby values represent the mean. Sample sizes for each category are provided in the bottom of the plot; **c** Percentage of significantly associated enhancer-enhancer pairs (y-axis) per number of enhancers significantly associated with the gene (x-axis); **d** Percentage of genes in which enhancer-enhancer pairs are significantly associated (y-axis) per number of genes in which they were tested (x-axis). A total of 45,679 distinct enhancer-enhancer pairs were analysed. For all boxplots, the length of the box corresponds to the IQR with the centre line corresponding to the median, the upper and lower whiskers represent the largest or lowest value no further than 1.5 × IQR from the third and first quartile, respectively.

A key question in enhancer biology is whether enhancers regulate genes in isolation or in simultaneous concert with other enhancers. Enhancer co-activity measurements in single cells can give clues about the cooperativity of enhancers in gene regulation. In our approach, the number of active enhancer combinations observed in a single cell depends on the total number of nearby enhancers associated with a gene. For instance, the gene *ABHD4* has three nearby associated enhancers, and we observed all seven combinations of enhancers active in at least a single cell (Fig. 2a). The seven combinations comprise (i) three combinations of only one enhancer active in a cell, (ii) three combinations of two enhancers active in the same cell and (iii) one combination with all three enhancers active in the same cell (Fig. 2a).

To understand the upper limit in identifying enhancer co-activity in this single cell multimodal dataset, for each gene, we measured the percentage of enhancer combinations observed in at least one cell, as in the example of Fig. 2a in which 7 combinations (100% of all possible combinations) were observed. We found that on average 76.3% of all possible combinations of enhancers across the 5087 genes are observed in at least one cell (Fig. 2b). This indicates that certain combinations of enhancer co-activity either do not occur in the cells or cannot be detected in this single cell dataset. However, we note that the percentage of observed enhancer combinations is largely dependent on the total number of enhancers associated with the gene (Spearman R = −0.89, *p*-value < 2.2e^−308^, Fig. 2b). For instance, between 95.3% and 98.2% of all enhancer combinations are observed for genes with up to 4 enhancers, whereas only 8.6% of combinations were observed for genes with 10–15 associated enhancers. This decrease is expected, since the number of possible combinations increases exponentially, with 1023 distinct combinations possible for genes with 10 associated enhancers. Next, we evaluated the number of enhancer-enhancer pairs that have statistically significant associations (correlation > 0.05 and FDR < 5%). Note that the number of possible enhancer pairs is lower than the number of enhancer combinations (e.g. 45 possible enhancer pairs with 10 associated enhancers). We observed that the majority of possible enhancer pairs are significantly associated, with between 67.0% and 79.6% of the possible enhancer-enhancer pairs significantly associated (Fig. 2c, Supplementary Fig. 7), depending on the number of associated enhancers per gene (Spearman R = −0.35, *p*-value = 1.3e^−153^). The high levels of significant enhancer associations in genes with 10 or more enhancers suggests considerable cooperativity between enhancers.

When taking an enhancer-centric perspective, we find that enhancer pairs are more likely to associate with a higher proportion of genes when more genes are present in their vicinity, with as much as 94.5% genes being significantly associated with an enhancer pair when 10 to 15 genes are present in its vicinity (Fig. 2d, Spearman R = 0.3, *p*-value < 2.2e^−308^). This illustrates the presence of genomic regions with high enhancer and gene activity and the high sharing of enhancers across genes, as previously observed^25^. For instance, two enhancers in chr6 (chr6:26104800-26105400 and chr6:26189200-26191000) display significant associations with 21 out of 22 of their neighbouring genes, many of which are found within co-expression gene clusters encoding for Histone proteins^12,27^ (Supplementary Data 4). Similar patterns of enhancer co-activity are observed when restricting the maximum distance between genes and enhancers to 200 kb instead of 1 Mb (15,130 enhancer-enhancer associations, Supplementary Fig. 8). In summary, we found that enhancer co-activity is highly prevalent across genes, occurring between the majority of enhancers associated with the same gene. This co-activity of enhancers in the same single cells suggests that enhancers do not act in isolation, but rather as a group, possibly functioning as shadow enhancers.

### Molecular features of co-active enhancers

**Fig. 3:**
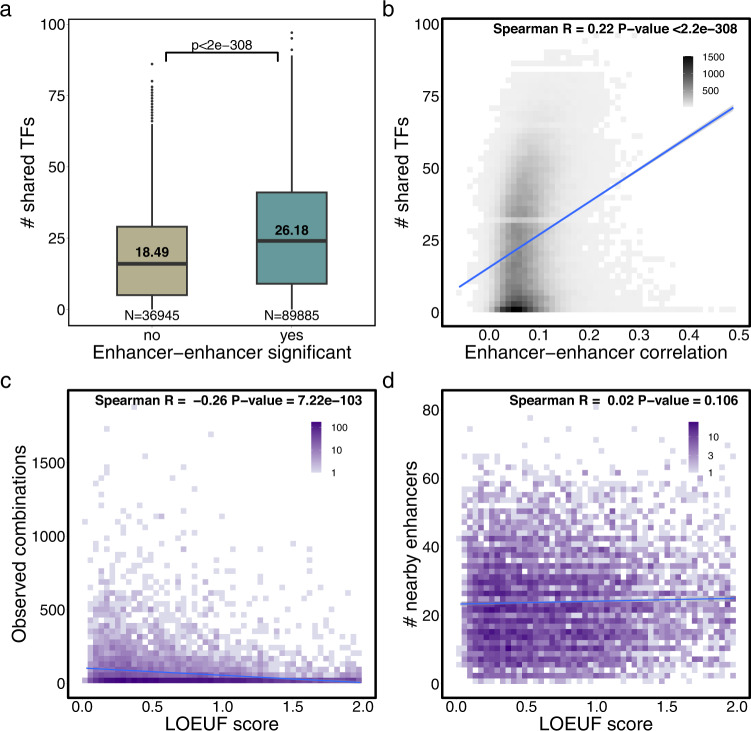
Features of enhancer co-activity. **a** Number of distinct TFs with binding sites (ReMap data) in both enhancers of an enhancer-enhancer pair (shared TFs), depending on their association significance. Two-tailed Wilcoxon test *p*-value < 2.2e^−308^. The length of the box corresponds to the IQR with the centre line corresponding to the median, the upper and lower whiskers represent the largest or lowest value no further than 1.5 × IQR from the third and first quartile, respectively. Values above the median line represent the mean; **b** Number of shared TFs per enhancer-enhancer correlation coefficient (*N* = 126,830); **c** Number of enhancer combinations observed in at least one cell (y-axis) per gene LOEUF score (x-axis) (*N* = 6895); **d** Number of enhancers within 1 Mb of the gene TSS (regardless of gene-enhancer association significance) per gene LOEUF score (*N* = 6895). Fit lines represent a linear regression model.

Having sets of co-active enhancers per gene enables the analysis of their properties as a group. We first explored the concordance in transcription factor (TF) binding in co-active enhancers. For this, we overlapped enhancer regions with LCL transcription factor binding sites from ReMap (ChIP-seq data)^28^, obtaining 417,099 enhancer-TF pairs. We then measured the number of distinct TFs with binding sites present in both enhancers of an enhancer-enhancer pair (see Methods). We found that the 89,885 significantly associated enhancer pairs shared higher numbers of TFs (mean = 26.2) than non-significant enhancer pairs (mean = 18.5, Wilcoxon test *p*-value < 2.2e^−308^, Fig. 3a). Moreover, we found that higher enhancer-enhancer correlations correspond to higher number of shared TFs (Spearman R = 0.22, *p*-value < 2.2e^−308^, Fig. 3b). This trend was confirmed when measuring the Jaccard similarity index (JI) between the sets of TFs binding both enhancers in the pair (Spearman R = 0.15, *p*-value < 2.2e^−308^, Supplementary Fig. 9a). As we found that significantly correlated enhancers were found at moderately lower genomic distances than non-significant enhancer pairs (mean absolute distance significant = 466.6 kb, non-significant = 486.5 kb, Supplementary Fig. 10), the TF sharing results could be affected by distance. However, we still observe higher TF sharing in 15,242 distance-matched significant (mean = 23.3) and non-significant enhancer pairs (mean = 15.8, Wilcoxon test *p*-value = 7.7e^−267^, Supplementary Fig. 9b). Importantly, all these results were replicated when using TF data from the MotifMap dataset^29^, which is based on genome sequence scans for known TF motifs and thus is not cell-type specific (Supplementary Fig. 11). Highly similar patterns were observed with the MotifMap dataset when considering an independent dataset comprising 13,311 multimodal PBMC single cells (Supplementary Fig. 12). Finally, similar results were observed when considering ABC model gene-enhancer links and LCL ReMap^28^ TF data (e.g. Spearman R = 0.33, *p*-value < 2.2e^−308^ for the number of shared TFs per enhancer-enhancer correlation, Supplementary Fig. 13).

Previous studies demonstrated that the number and size of enhancers regulating a gene increases with the gene’s essentiality^7^. We explore this in the context of enhancer-enhancer associations using gnomAD LOEUF scores^30^. For this, we compared the number of significantly associated enhancers and gene essentiality (lower LOEUF scores indicate higher essentiality). We found that gene essentiality is negatively correlated with the number of (i) enhancer-enhancer combinations observed in at least one single cell (Spearman R = −0.26, *p*-value = 7.2e^−103^, Fig. 3c) and (ii) significant enhancer-enhancer pairs (Spearman R = −0.23, *p*-value = 1.2e^−84^, Supplementary Fig. 14a). This indicates that the more essential a gene is, the more enhancer combinations regulate it. Interestingly, the number of nearby enhancers (regardless of significance) did not correlate with gene essentiality (Spearman R = 0.02, *p*-value = 0.11, Fig. 3d), suggesting that only significantly associated enhancers and their combinations are relevant. Next, we considered enhancer-domain scores from Wang & Goldstein 2020^7^, which reflect the redundancy of a gene’s non-coding regulatory architecture and correlate with gene essentiality. We found significant positive correlation between enhancer-domain scores (higher scores indicate higher redundancy) and the number of (i) enhancer combinations (Spearman R = 0.1, *p*-value = 1.1e^−15^) and (ii) significant enhancer pairs (Spearman R = 0.08, *p*-value = 1.0e^−10^, Supplementary Fig. 14b, c). A negative correlation was observed against the total number of nearby enhancers (Spearman R = −0.08, *p*-value = 5.5e^−11^, Supplementary Fig. 14d). Importantly, increased TF sharing in associated enhancer pairs and an increase in gene essentiality with higher number of enhancer pairs was also found when considering more stringent enhancer-enhancer association significance thresholds (e.g. correlation > 0.1, Supplementary Fig. 15, Supplementary Data 5), and when restricting maximum distance between gene and enhancers to 200 kb instead of 1 Mb (Supplementary Fig. 16). These results highlight the importance of robust gene expression regulation through shadow enhancers in essential genes.

## Discussion

While much is known regarding transcription regulation and the potential for multiple (shadow) enhancers to regulate a certain gene^7–9,31^, it is currently unknown whether these multiple enhancers are active at the same time, as this information cannot be obtained from bulk tissue measurements. Our work proposes the use of multimodal single cell RNA-seq and ATAC-seq^22^ in the same cells to study the co-activity of enhancers in gene expression regulation. Indeed, by having information of enhancer and gene activity in the same single cell, we defined sets of enhancers active upon gene expression, and suggest that enhancer co-activity occurs pervasively across genes. Overall, we found that the set of enhancers that are active upon gene expression can be highly dynamic, with cells presenting disparate patterns of enhancer activity. It is likely that enhancer redundancy serves to drive stable and precise gene transcription, robust to genetic variation and environmental stress^32^. By finding higher numbers of co-active enhancers in essential genes – as well as extensive sharing of transcription factor binding in co-active enhancers – our study corroborates this role of enhancer redundancy. Indeed, we complement previous studies which found a relationship between the number of conserved nucleotides in enhancers and gene essentiality^7^ by showing that this extends to co-active enhancer combinations.

A key limitation of our proof-of-principle study is the exploration of a single cell line (LCL). Further studies across cell types and tissues are needed to reproduce these results and demonstrate enhancer co-activity pervasiveness. Moreover, our study is based on correlation measurements from sparse single cell data, which does not provide concrete evidence of enhancer-enhancer interactions. Orthogonal approaches and experimental evidence is required to confirm their validity and understand whether these predicted enhancer pairs interact and have functional relevance. Although single cell data proves useful in connecting gene expression and regulatory element activity^22,33–35^, its usage for determining enhancer co-activity may entail several limitations. First, while we try to expose the breadth of enhancer co-activity that could occur in single cells (e.g. observed enhancer combinations), not finding certain combinations of enhancers in at least one cell does not mean they do not occur, since single cell technology – even when considering >20,000 cells – may not identify co-active enhancers below certain detection levels. Moreover, some enhancer regulatory patterns may only be revealed under particular cellular contexts or stresses, as has been demonstrated in recent studies of context-dependent quantitative trait loci (QTL) and reporter assays^36–40^. The use of larger datasets of multimodal single cell data, as well as exploring context-dependent effects and other cell-types would likely allow us to observe more enhancer activity combinations. On the other hand, enhancer pair co-activity – even with significant correlation – does not necessarily imply that the two enhancers are active in regulating the same gene or acting together. Indeed, enhancer co-activity could occur as a consequence of the chromatin being open, which in itself could occur stochastically or due to regulation of other nearby genes. Given the high levels of co-expression found between nearby genes^12^ and the sharing of enhancers between co-expressed genes we previously observed^25^, enhancer co-activity is likely influenced by local gene co-expression. In addition, given the sparsity inherent to single cell data, in particular in scATAC-seq, where only two DNA molecules are assessed, gene-enhancer and enhancer-enhancer association analysis through correlation may have high false positive rates. We attempted to address this by analysing correlation results with varying *p*-value and correlation level stringencies, and by reproducing results in independent multimodal datasets. Finally, while gene expression and enhancer activity in the same cell are indicative of their relationship, gene transcription is a highly dynamic process and could be partially decoupled in time with nearby enhancer activation^41^, i.e. there could be time lags between enhancer activity and the expression of targeted genes, which would decrease our ability to detect their correlation across single cells.

To exploit known biological knowledge we limited our analysis of enhancer-enhancer co-activity to known enhancer regions from the EpiMap resource^19^ in the same cell line for which single cell data was available^22^ (GM12878 LCL). While this allowed us to perform a more focused analysis, we cannot exclude the fact that other genomic regions with enhancer potential were omitted from analysis. In fact, many ATAC-seq peaks in the single cell data fall outside enhancer regions and could be further exploited for regulatory element identification and correlation, as performed in other studies^22,42^. Although we provided an overview of the potential relationship between the several enhancers targeting the same gene, further study is required to understand whether these physically interact, in a synergistic or even repressive way towards gene expression. Indeed, while we found higher bulk Hi-C contacts between co-active enhancers, finding correlation between enhancer activity does not provide information on their physical interaction and it is yet unclear if all co-active enhancers interact with each other as well as the gene promoter in a single cell. Further studies with multi-omics single cell datasets, including Hi-C and massively parallel reporter assays, are poised to address these questions in the near future^43,44^.

An improved understanding of enhancer biology would aid the interpretation of non-coding genetic variants. For instance, estimating the robustness of genes to regulatory region mutations can explain why single mutations in their enhancers have little or no effect. Approaches such as regulatory region mutation burden^7,45^ may perform differently depending on the gene regulatory redundancy. In our study, we provide a proof-of-principle framework to define sets of relevant enhancers per gene which can be exploited in gene-trait association testing. Knowing the exact regulatory element circuitry for each gene and accounting for enhancer redundancy is expected to improve the use of whole genome sequencing in the discovery of novel disease genes and in disease diagnosis.

## Methods

### SHARE-seq single cell data

The single cell dataset used in the study was obtained from Ma et al. 2020^22^ through GEO (GSE140203). This consisted of preprocessed gene expression counts and ATAC-seq peaks from the single cell SHARE-seq method for the GM12878 lymphoblastoid cell line (LCL). The original dataset included 26,434 genes expressed across 26,589 cells (GSM4156603, rep3, cells with >300 and <7.500 genes expressed) and 507,307 ATAC-seq peaks across 67,418 cells^22^ passing quality control (GSM4156592, rep3). On this dataset, we added genomic coordinates (hg19) and Ensembl gene IDs from Gencode v19^46^. We excluded non-protein coding genes, as well as genes in non-autosomes or in the major histocompatibility complex region (MHC, chr6:29500000-33600000). We binarised both the gene expression matrix and ATAC-seq peaks data (values > 1 became 1, values = 0 remained 0).

### PBMC single cell data

Public multimodal single cell data on peripheral blood mononuclear cells (PBMCs) was obtained from 10x Genomics (https://www.10xgenomics.com/resources/datasets/pbmc-from-a-healthy-donor-granulocytes-removed-through-cell-sorting-10-k-1-standard-1-0-0). For data processing and quality-control (QC), the Signac tutorial on analysing this dataset was followed (https://github.com/stuart-lab/signac/blob/master/vignettes/pbmc_multiomic.Rmd). Briefly, cells that are outliers for number of ATAC-seq fragments, RNA-seq counts, NucleosomeSignal or TSSEnrichment (Signac package^47^) were excluded. ATAC-seq peaks were identified with MACS2^48^. After QC, 11,331 cells with scRNA-seq and scATAC-seq were used for analysis. All cell types were used. We identified gene-enhancer associations and enhancer-enhancer associations as for the SHARE-seq dataset (described below), but using enhancer annotations from a mononuclear blood cell line from the EpiMap repository^19^ (BLD.MONONUC_BSS01279, 33,776 distinct non-overlapping enhancers) and keeping only cases with positive gene-enhancer correlation. ATAC-seq peak coordinates were converted to hg19 with the UCSC liftOver tool. As with the SHARE-seq dataset, only protein coding genes in autosomes were considered.

### Gene-enhancer associations

Gene-enhancer association predictions in the GM12878 LCL were obtained from our previous work at Ribeiro et al. 2022^25^, Supplementary Data 4. These were identified with the SHARE-seq dataset^22^ described above, and also used to identify enhancer-enhancer associations. Briefly, we utilised processed and quality-controlled ATAC-seq peaks from Ma et al. 2020^22^ (GSM4156592, rep3). We considered the subset of 24,844 cells that also had gene expression measurements (GSM4156603, rep3). Next, we retained ATAC-seq peaks overlapping GM12878-specific enhancer annotations from the EpiMap repository^19^ (hg19). For this, we considered the EnhG1, EnhG2, EnhA1, EnhA2 states from the 18-state chromHMM models, which refer to “active enhancers” and “genic enhancers” (https://egg2.wustl.edu/roadmap/web_portal/chr_state_learning.html#exp_18state). The overlap was performed with bedtools (v2.29.2) intersect with the -F0.5 parameter (i.e. requiring ≥ 50% of the peak to be inside the enhancer). Book-ended EpiMap enhancer annotations were previously merged using bedtools merge with default parameters (leading to 33,776 distinct enhancer regions). Finally, we integrated gene expression and open chromatin activity measurements (binarised) for the same cells and enhancer regions within ±1 Mb of a gene TSS were tested for association with the gene through Pearson correlation (equivalent to Spearman correlation when using binary data), in a total of 350,182 tests performed (17,300 distinct enhancers, 16,463 distinct genes). We only considered protein-coding genes in autosomal chromosomes. For each test, we shuffled the expression vector of the gene 1000 times and recalculated the correlation. We then derive an empirical p-value for the probability that the observed value is more extreme than the randomised correlations. To control for the total number of tests we applied the Benjamini–Hochberg procedure for FDR on the empirical *p*-values. We determined gene-enhancer pairs with correlation coefficient >0.05 and FDR < 5% as significant gene-enhancer associations (total of 32,883 associations between 7551 distinct enhancers and 6944 distinct genes).

### Enhancer-enhancer associations

We identified enhancer-enhancer associations through a gene-centric approach, using the 24,844 cells from Ma et al. 2020^22^ (rep3, GEO:GSM4156592, human LCL GM12878) with both scATAC-seq and scRNA-seq. For this, we started from the 32,883 gene-enhancer associations previously identified with the same 24,844 cells (described above). Then, for each gene, we (i) define the set of cells expressing the gene, which is used as the background of the association test, (ii) for each enhancer associated with the gene, we define the set of cells in which the enhancer is active, (iii) for each pair of enhancers in the set of associated enhancers, we perform Pearson correlation between the enhancer activity vectors across the set of cells expressing the gene. These analyses were performed with custom R (v4.0.4) scripts (calculate_enh_enh_correlation.R, see Code Availability). We considered 6944 protein-coding genes with enhancer associations to 7551 enhancer regions, performing a total of 126,830 tests. To only consider robust correlation patterns, we excluded 34 genes which were expressed in less than 100 cells. We determined 89,885 enhancer-enhancer associations as significant by having a (i) Benjamini–Hochberg procedure FDR below 5% and (ii) an absolute Pearson correlation coefficient above 0.05, although other cutoffs were explored (Supplementary Fig. 1).

To compare enhancer-enhancer correlation levels between (i) enhancers significantly associated with genes and (ii) enhancers not associated with genes, we performed the same experiment described above, but considering all enhancers in the vicinity of genes (at most ±1 Mb away from the gene TSS), instead of only enhancers significantly associated with the gene. For this, 2,878,013 correlation tests were performed and the same significance cutoffs were applied to determine significant enhancer-enhancer associations. For result replication, enhancer-enhancer association tests were also performed for a biological replicate experiment (rep2, GEO:GSM4156591) containing 2788 cells with both scRNA-seq and scATAC-seq data. This provided us data to perform enhancer-enhancer correlation tests in rep2 for 79,788 out of the 126,830 gene-enhancer-enhancer combinations tested previously (in rep3).

To perform partial correlation accounting for gene expression, we used the R *ppcor* package (v1.0) for all 126,830 gene-enhancer-enhancer combinations previously tested. We used gene expression and enhancer activity across all 24,844 SHARE-seq cells, instead of considering only cells expressing the gene as previously. This is required since with binarised single cell data all cells expressing the gene have expression of 1, does having no variability. We reported the Pearson partial correlation (and *p*-values) between enhancer-enhancer combinations accounting for gene expression.

To compare our results with gene-enhancer definitions from other studies, we gathered 62,255 gene-enhancer association predictions from Nasser et al. 2021^17^ (ABC model, file: AllPredictions.AvgHiC.ABC0.015.minus150.ForABCPaperV3.txt) for the ‘GM12878-Roadmap’ cell type. Using this dataset, we measured ABC enhancer activity by overlapping SHARE-seq scATAC-seq data as described above. We could evaluate activity for 46,773 associations (10,862 distinct genes, 23,306 enhancer regions) out of 62,256 ABC gene-enhancer associations. From this data, we calculated enhancer-enhancer correlation as described above and compared multiple correlation and multiple test correction cutoffs to determine significance (Supplementary Fig. 1).

### Hi-C support of enhancer-enhancer associations

We obtained bulk Hi-C data for the GM12878 LCL cell line at 5 kb resolution from Rao et al. 2014^26^. We measured KR normalised (MAPQG0) contact between bins encompassing the midpoint of enhancer regions through custom Python v3.6.7 custom scripts. Normalised Hi-C contacts were log2-transformed. Missing data (enhancer-enhancer bins without Hi-C data) was replaced with 0. We then correlated Hi-C contacts with enhancer-enhancer activity correlation levels. To exclude the effect of distance in measuring Hi-C contacts, when indicated in the figure legend, we residualised the Hi-C contact levels for the distance between enhancer-enhancer pairs with a linear regression model. As a control, for each enhancer-enhancer pair, we produced another control pair composed of the first enhancer and an ‘enhancer’ region on the opposite up- or down-stream location in respect to the first enhancer midpoint (e.g. if the first enhancer is at position 5000 and the second at position 8000, the matching control region has the first enhancer at position 5000 but the second at position 2000).

### Transcription factor binding site analysis

We analysed two datasets of transcription factor binding sites (i) 4,052,293 binding sites (155 distinct TFs) based on ChIP-seq data collected from ReMap 2022^28^ for the human LCL GM12878 (hg19 assembly) and (ii) 4,474,877 binding sites (445 distinct TFs) based on motif mapping on hg19 from MotifMap^29^. For this last dataset, multiple motifs targeted by the same TFs were combined. To determine sharing of TF binding between pairs of enhancers, we first overlapped TF binding sites with enhancer regions using bedtools *intersect* with -F1 parameter (i.e. ensuring that 100% of the binding site is contained with the enhancer region coordinates). In this manner, we obtained 417,099 TF-enhancer combinations for the ReMap dataset and 46,112 combinations for the MotifMap dataset. For each dataset, we then counted the number of distinct TF with binding sites in both enhancers for each enhancer-enhancer pair analysed (TF sharing). Enhancer pairs without any TF binding site in the dataset were counted as having 0 shared TFs. To exclude a potential bias from the distance between enhancers in their likelihood of sharing TFs, we compared TF sharing between a set of enhancer-enhancer pairs matched for distance. For this, we matched 15,242 non-significant enhancer pairs to 15,242 subsampled significant enhancer pairs with a maximum absolute distance difference of 5% in between the enhancers. For instance, to match a non-significant enhancer pair apart for 1000 bp, we randomly sampled a significant enhancer pair with a distance between 950 and 1050 (1000 ± 1000 * 0.05).

### Gene essentiality analysis

To associate gene essentiality and number of enhancers associated with the gene, we obtained “loss-of-function observed/expected upper bound fraction” (LOEUF) scores per gene from gnomAD v2.1.1^30^ (https://gnomad.broadinstitute.org/). Low LOEUF scores indicate strong selection against predicted loss-of-function variation in a gene, i.e. higher predicted gene essentiality. We correlated LOEUF scores per gene with the number of observed or significant enhancer associations in 6895 genes with an attributed LOEUF score and at least one significantly associated enhancer. We performed the same analysis for enhancer-domain scores (EDS) obtained from Wang and Goldstein 2020^7^, which were available for 6925 genes.

### Statistics and reproducibility

Enhancer-enhancer associations were identified with public multimodal single cells, considering cells with both scRNA-seq and scATAC-seq. Results from two technical replicates (24,844 cells and 2788 cells) were compared. In addition, results from an additional dataset from a different cell line and multimodal technology (PBMC, 13,311 cells) were compared. Statistical analyses, including Wilcoxon tests were performed as two-tailed with R v4.0.4. Multiple test correction FDR was calculated across correlation tests using Benjamini–Hochberg procedure. All code to reproduce analyses is publicly available (see Code Availability).

### Reporting summary

Further information on research design is available in the Nature Portfolio Reporting Summary linked to this article.

## Supplementary information

Supplementary Information

Description of Additional Supplementary Files

Supplementary Data 1

Supplementary Data 2

Supplementary Data 3

Supplementary Data 4

Supplementary Data 5

Supplementary Data 6

Reporting Summary

## Data Availability

The enhancer-enhancer associations produced are available for download as Supplementary Data 1 to 5. Source data for Figs. 1 to 3 is provided as Supplementary Data 6. This and other source data is available in a Zenodo repository with the identifier: 10.5281/zenodo.7944850^49^. Input data used in this study is available in the public domain. LCL single cell RNA-seq and ATAC-seq (SHARE-seq) processed data is publicly available through GEO (accession: GSE140203). Peripheral blood mononuclear cells (PBMCs) multimodal data was obtained from 10x Genomics (https://www.10xgenomics.com/resources/datasets/pbmc-from-a-healthy-donor-granulocytes-removed-through-cell-sorting-10-k-1-standard-1-0-0).

## Code availability

Code to produce enhancer-enhancer associations and all figures in the manuscript is provided in https://github.com/diogomribeiro/enhEnh and deposited in the linked Zenodo repository with the identifier: 10.5281/zenodo.7944850^49^.

## References

[1] Andersson R, Sandelin A (2020). Determinants of enhancer and promoter activities of regulatory elements. Nat. Rev. Genet..

[2] Strober BJ (2019). Dynamic genetic regulation of gene expression during cellular differentiation. Science.

[3] Kolovos P, Knoch TA, Grosveld FG, Cook PR, Papantonis A (2012). Enhancers and silencers: an integrated and simple model for their function. Epigenetics Chromatin.

[4] Claringbould A, Zaugg JB (2021). Enhancers in disease: molecular basis and emerging treatment strategies. Trends Mol. Med..

[5] Lee TI, Young RA (2013). Transcriptional regulation and its misregulation in disease. Cell.

[6] Panigrahi A, O’Malley BW (2021). Mechanisms of enhancer action: the known and the unknown. Genome Biol..

[7] Wang X, Goldstein DB (2020). Enhancer domains predict gene pathogenicity and inform gene discovery in complex disease. Am. J. Hum. Genet..

[8] Waymack R, Fletcher A, Enciso G, Wunderlich Z (2020). Shadow enhancers can suppress input transcription factor noise through distinct regulatory logic. Elife.

[9] Lam DD (2015). Partially redundant enhancers cooperatively maintain Mammalian pomc expression above a critical functional threshold. PLoS Genet.

[10] Osterwalder M (2018). Enhancer redundancy provides phenotypic robustness in mammalian development. Nature.

[11] Delaneau, O. et al. Chromatin three-dimensional interactions mediate genetic effects on gene expression. *Science* **364**, eaat8266 (2019). 10.1126/science.aat8266 31048460

[12] Ribeiro DM (2021). The molecular basis, genetic control and pleiotropic effects of local gene co-expression. Nat. Commun..

[13] Avalos D (2023). Genetic variation in cis-regulatory domains suggests cell type-specific regulatory mechanisms in immunity. Commun. Biol..

[14] Kvon EZ, Waymack R, Gad M, Wunderlich Z (2021). Enhancer redundancy in development and disease. Nat. Rev. Genet..

[15] Miesfeld JB (2020). The remote enhancer provides transcriptional robustness during retinal ganglion cell development. Proc. Natl Acad. Sci. USA.

[16] Levine M (2010). Transcriptional enhancers in animal development and evolution. Curr. Biol..

[17] Nasser J (2021). Genome-wide enhancer maps link risk variants to disease genes. Nature.

[18] Fulco CP (2019). Activity-by-contact model of enhancer-promoter regulation from thousands of CRISPR perturbations. Nat. Genet..

[19] Boix CA, James BT, Park YP, Meuleman W, Kellis M (2021). Regulatory genomic circuitry of human disease loci by integrative epigenomics. Nature.

[20] Kumasaka N, Knights AJ, Gaffney DJ (2019). High-resolution genetic mapping of putative causal interactions between regions of open chromatin. Nat. Genet..

[21] Chen X (2021). Tissue-specific enhancer functional networks for associating distal regulatory regions to disease. Cell Syst..

[22] Ma S (2020). Chromatin potential identified by shared single-cell profiling of RNA and chromatin. Cell.

[23] Hao Y (2021). Integrated analysis of multimodal single-cell data. Cell.

[24] Cao J (2018). Joint profiling of chromatin accessibility and gene expression in thousands of single cells. Science.

[25] M Ribeiro D, Ziyani C, Delaneau O (2022). Shared regulation and functional relevance of local gene co-expression revealed by single cell analysis. Commun. Biol..

[26] Rao SSP (2014). A 3D map of the human genome at kilobase resolution reveals principles of chromatin looping. Cell.

[27] Marzluff WF, Gongidi P, Woods KR, Jin J, Maltais LJ (2002). The human and mouse replication-dependent histone genes. Genomics.

[28] Hammal F, de Langen P, Bergon A, Lopez F, Ballester B (2022). ReMap 2022: a database of Human, Mouse, Drosophila and Arabidopsis regulatory regions from an integrative analysis of DNA-binding sequencing experiments. Nucleic Acids Res..

[29] Daily K, Patel VR, Rigor P, Xie X, Baldi P (2011). MotifMap: integrative genome-wide maps of regulatory motif sites for model species. BMC Bioinforma..

[30] Karczewski KJ (2020). The mutational constraint spectrum quantified from variation in 141,456 humans. Nature.

[31] Hay D (2016). Genetic dissection of the α-globin super-enhancer in vivo. Nat. Genet..

[32] Barolo S (2012). Shadow enhancers: frequently asked questions about distributed cis-regulatory information and enhancer redundancy. Bioessays.

[33] Wang C (2020). Integrative analyses of single-cell transcriptome and regulome using MAESTRO. Genome Biol..

[34] Li G (2022). A deep generative model for multi-view profiling of single-cell RNA-seq and ATAC-seq data. Genome Biol..

[35] Ranzoni AM (2021). Integrative single-cell RNA-Seq and ATAC-Seq analysis of human developmental hematopoiesis. Cell Stem Cell.

[36] Nathan A (2022). Single-cell eQTL models reveal dynamic T cell state dependence of disease loci. Nature.

[37] Perez RK (2022). Single-cell RNA-seq reveals cell type-specific molecular and genetic associations to lupus. Science.

[38] Yazar S (2022). Single-cell eQTL mapping identifies cell type-specific genetic control of autoimmune disease. Science.

[39] Soskic B (2022). Immune disease risk variants regulate gene expression dynamics during CD4 T cell activation. Nat. Genet..

[40] Santiago-Algarra D (2021). Epromoters function as a hub to recruit key transcription factors required for the inflammatory response. Nat. Commun..

[41] Li, C., Virgilio, M., Collins, K. L. & Welch, J. D. Single-cell multi-omic velocity infers dynamic and decoupled gene regulation. *bioRxiv*10.1101/2021.12.13.472472 (2021).

[42] Bravo González-Blas, C. et al. SCENIC+: single-cell multiomic inference of enhancers and gene regulatory networks. *bioRxiv*10.1101/2022.08.19.504505 (2022). 10.1038/s41592-023-01938-4 PMC10482700 37443338

[43] Zhang R, Zhou T, Ma J (2022). Multiscale and integrative single-cell Hi-C analysis with Higashi. Nat. Biotechnol..

[44] Sahu B (2022). Sequence determinants of human gene regulatory elements. Nat. Genet..

[45] Bocher O, Génin E (2020). Rare variant association testing in the non-coding genome. Hum. Genet..

[46] Frankish A (2021). GENCODE 2021. Nucleic Acids Res..

[47] Stuart T, Srivastava A, Madad S, Lareau CA, Satija R (2021). Single-cell chromatin state analysis with Signac. Nat. Methods.

[48] Zhang Y (2008). Model-based analysis of ChIP-Seq (MACS). Genome Biol..

[49] Ziyani, C., Delaneau, O. & Ribeiro, D. M. Multimodal single cell analysis infers widespread enhancer co-activity in a lymphoblastoid cell line. *Zenodo*10.5281/zenodo.7944850 (2023). 10.1038/s42003-023-04954-4 PMC10219981 37237005

