# Supplementary material for: Multimodal single cell analysis infers widespread enhancer co-activity in a lymphoblastoid cell line
Source: Commun Biol. 2023 May 26;6:563. doi: 10.1038/s42003-023-04954-4 (PMC10219981; doi:10.1038/s42003-023-04954-4)
Supplement: Supplementary file 1 — Supplementary Information [file 42003_2023_4954_MOESM1_ESM.pdf]

Supplementary Information

Multimodal single cell analysis infers widespread enhancer co-activity in a lymphoblastoid cell line

Chaymae Ziyani[1,2], Olivier Delaneau[1,2], Diogo M. Ribeiro[1,2*]

[1]Department of Computational Biology, University of Lausanne, Lausanne, Switzerland,

[2]Swiss Institute of Bioinformatics (SIB), Lausanne, Switzerland

*corresponding author: diogo.ribeiro@unil.ch

Supplementary Figures 1 to 16

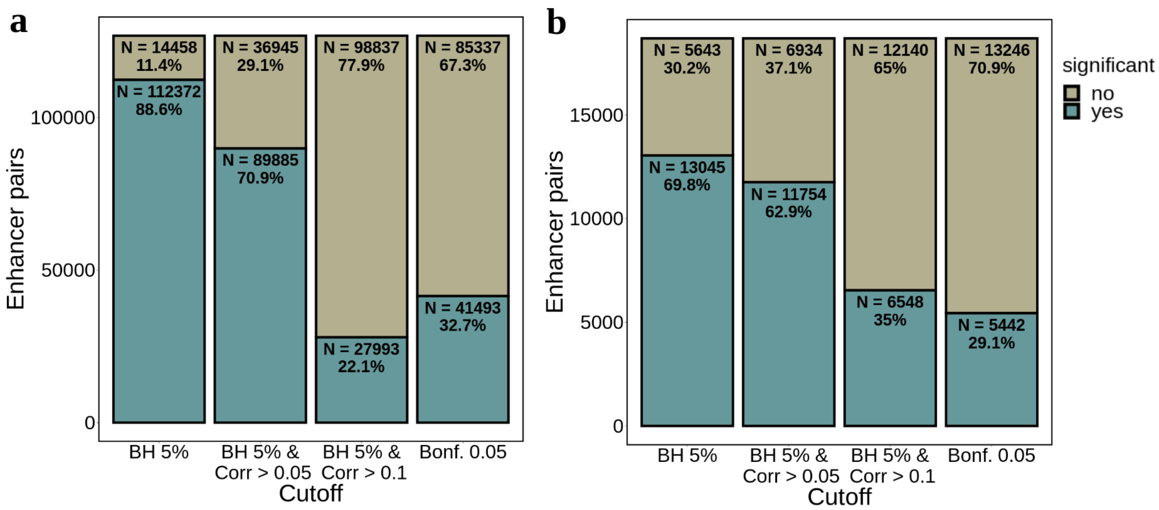

Supplementary Figure 1 Percentage of significantly enhancer-enhancer associations depending on the multiple testing correction and correlation coefficient cutoff used. a enhancer models from EpiMap and gene-enhancer associations based on SHARE-seq single cell data (N = 126,830); **b** enhancer models and gene-enhancer associations based on the ABC model (N = 20,611).

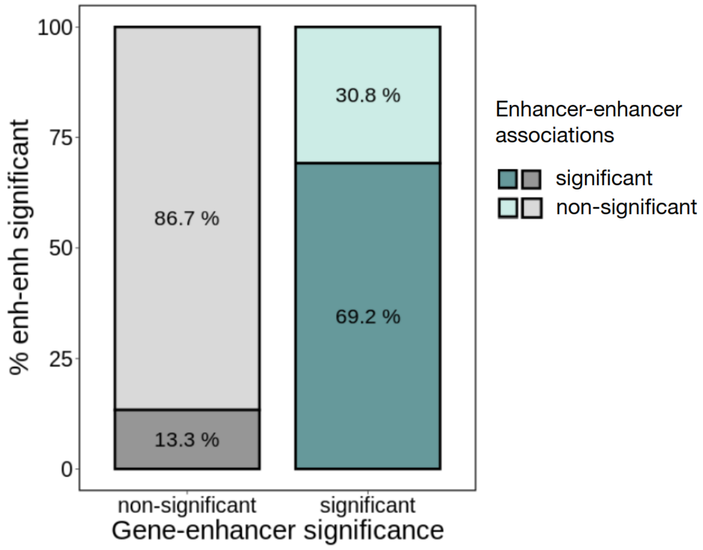

Supplementary Figure 2 Percentage of significantly enhancer-enhancer associations depending on the significance of the underlying gene-enhancer associations. The gene-enhancer *significant* category refers to cases in which both enhancers are significantly associated with the gene. Conversely, the gene-enhancer *non-significant* category refers to cases where neither of the enhancers is significantly associated with the gene. Results for cases where one enhancer is significantly associated with the gene but the other enhancer is not are not shown. Note that a slightly lower percentage of significant enhancer-enhancer associations (70.9% to 69.2% with significant gene-enhancer associations) is due to the higher burden of multiple test correction in this analysis (N = 2,878,013).

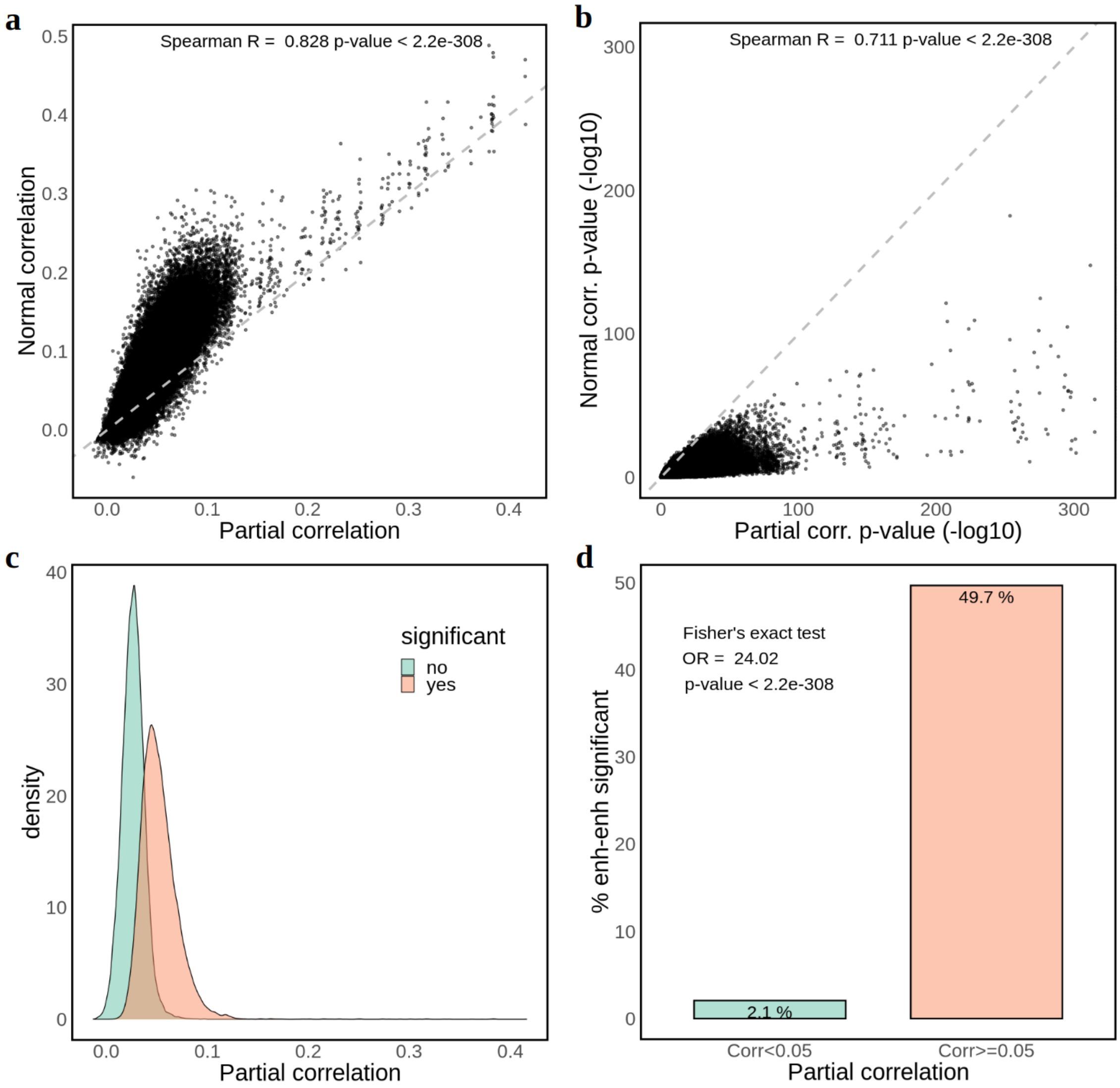

Supplementary Figure 3 Enhancer-enhancer correlation accounting for gene expression a comparison between correlation coefficients between partial correlation accounting for gene expression and our previous approach (N = 126,830)**; b** comparison between -log10 correlation p-values (N = 126,830)**; c** partial correlation coefficients depending on significant/non-significant definition from previous approach (significant N = 89,885, non-significant N = 36,945)**; d** percentage of enhancer pairs defined as significant depending on a partial correlation threshold. Results of a two-tailed Fisher's exact test are shown.

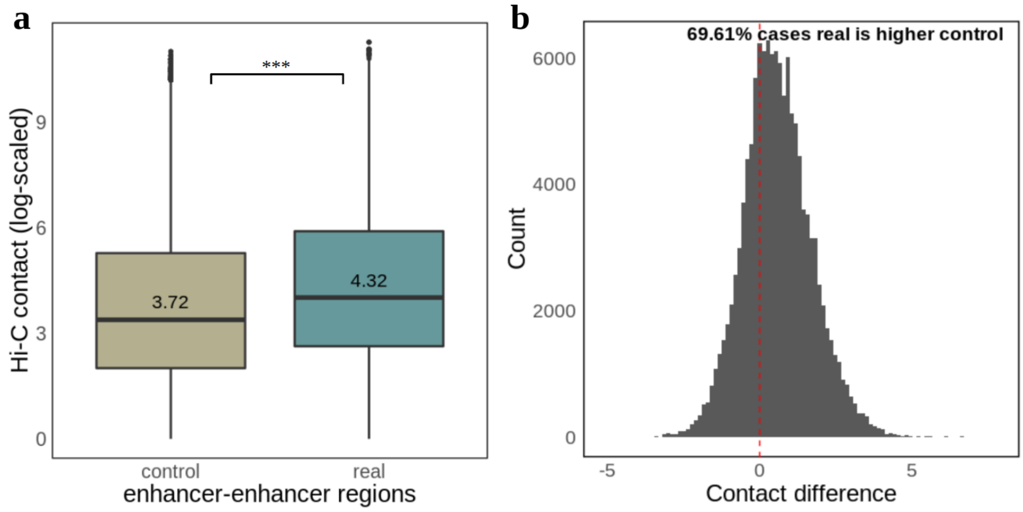

Supplementary Figure 4 Comparison of Hi-C contacts between real enhancer-enhancer coordinates and distance-matched control regions. a boxplot of Hi-contacts (log-scaled) for real enhancer-enhancer coordinates and control regions, in which the coordinates for one enhancer (enhancer 1) are kept, but for the other enhancer are replaced with the upstream/downstream position (see Methods). The length of the box corresponds to the IQR with the centre line corresponding to the median, the upper and lower whiskers represent the largest or lowest value no further than 1.5 × IQR from the third and first quartile, respectively. Values above the median line represent the mean. "***" denotes two-tailed Wilcoxon test p-value < $2.2e^{-308}$; **b** difference in contact intensities between real and control regions. A shift of the distribution to the right (above 0) represents higher Hi-C contacts in the real regions compared to control. The midpoint position of the enhancer start and end coordinates was used to calculate Hi-C contacts (N = 126830). Hi-C contact missing data (3.1% of the cases) was replaced with 0.

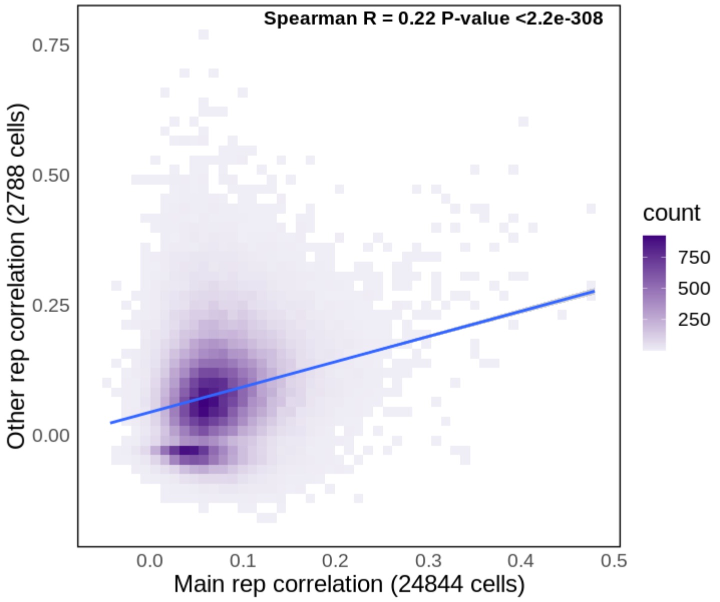

Supplementary Figure 5 Enhancer-enhancer association correlation values between two biological replicates. Gene-enhancer-enhancer associations tested in rep3 (main rep, 24844 cells, x-axis) were tested for correlation using data from rep2 (other rep, y-axis, 2788 cells). A total of 79,788 gene-enhancer-enhancer associations had sufficient data to be compared (e.g. gene expression in at least 100 cells and ATAC-seq data overlapping the enhancers).

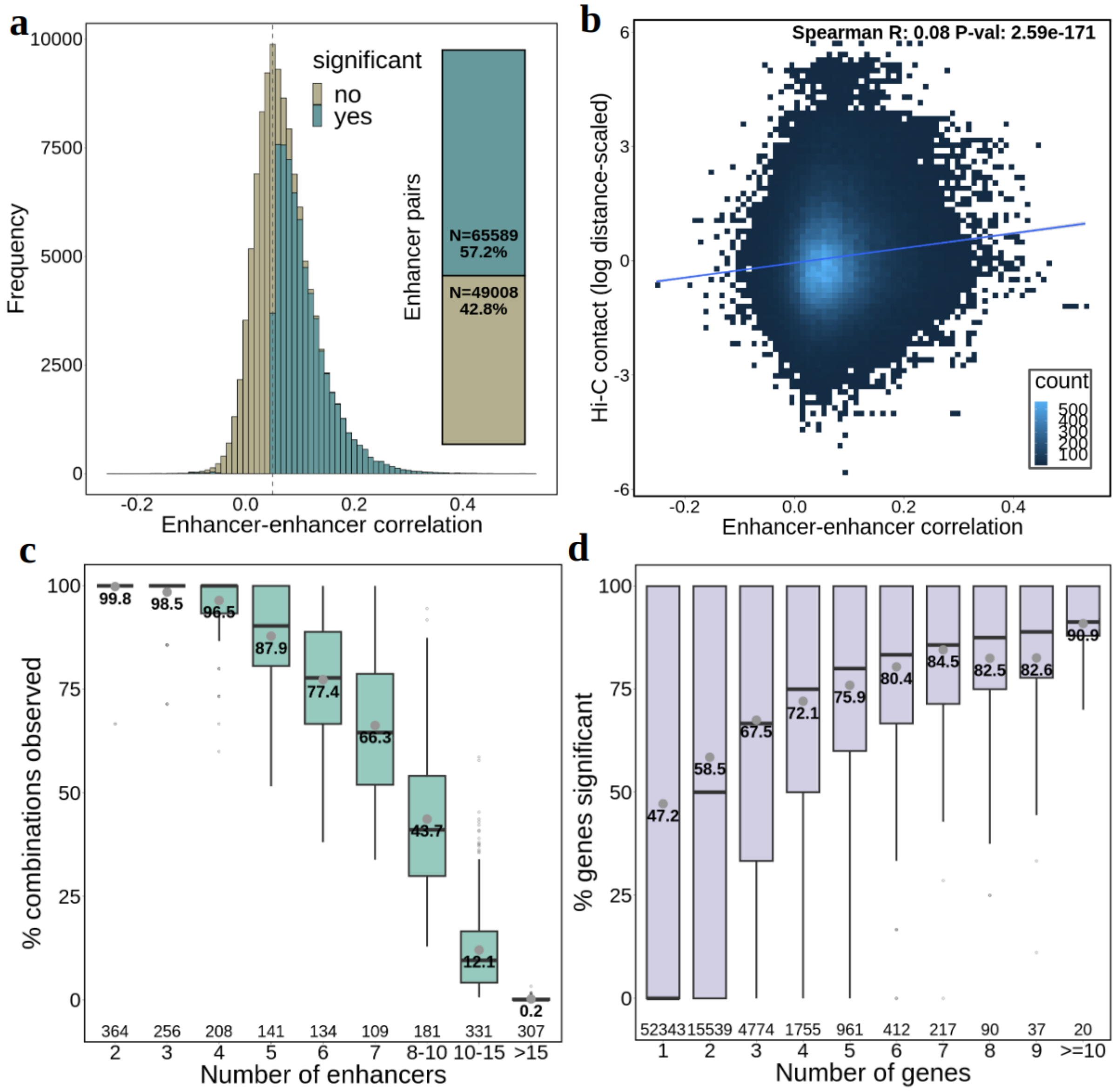

Supplementary Figure 6 Enhancer co-activity in the PBMC multimodal dataset. a enhancer-enhancer association correlation distribution (N = 114,597). The inner plot denotes the percentage of significant associations (green colour, FDR < 5% and absolute correlation > 0.05); **b** Hi-C contacts (log distance-scaled, 5kb resolution, GM12878) per enhancer-enhancer correlation value (N = 114,597); **c** percentage of enhancer combinations observed in at least one cell (y-axis) per number of enhancers significantly associated with the gene (x-axis). Grey dots and nearby values represent the mean. Sample sizes for each category are provided in the bottom of the plot; **d** percentage of genes in which enhancer-enhancer pairs are significantly associated (y-axis) per number of genes in which they were tested (x-axis). For all boxplots, the length of the box corresponds to the IQR with the centre line corresponding to the median, the upper and lower whiskers represent the largest or lowest value no further than 1.5 × IQR from the third and first quartile, respectively.

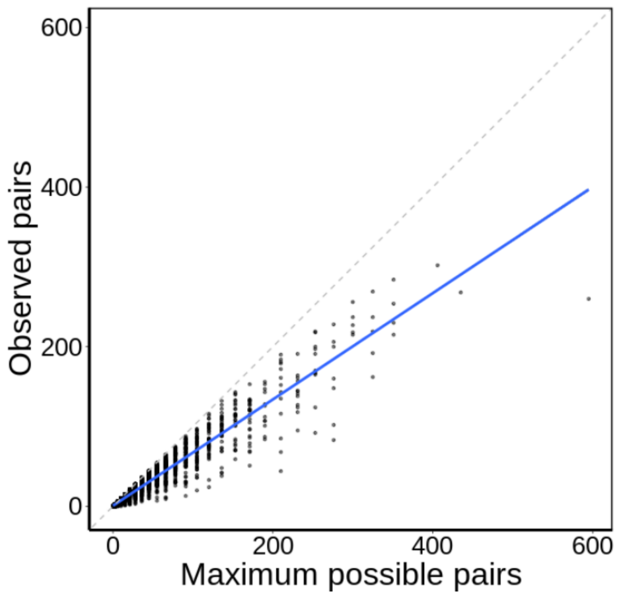

Supplementary Figure 7 Comparison between observed and maximum possible enhancer-enhancer pairs across genes. Each dot represents a gene. The number of maximum possible pairs scales with the number of enhancers (significantly associated) per gene. The number of observed pairs refers to significant association between the enhancer pair. Fit line corresponds to a linear regression model.

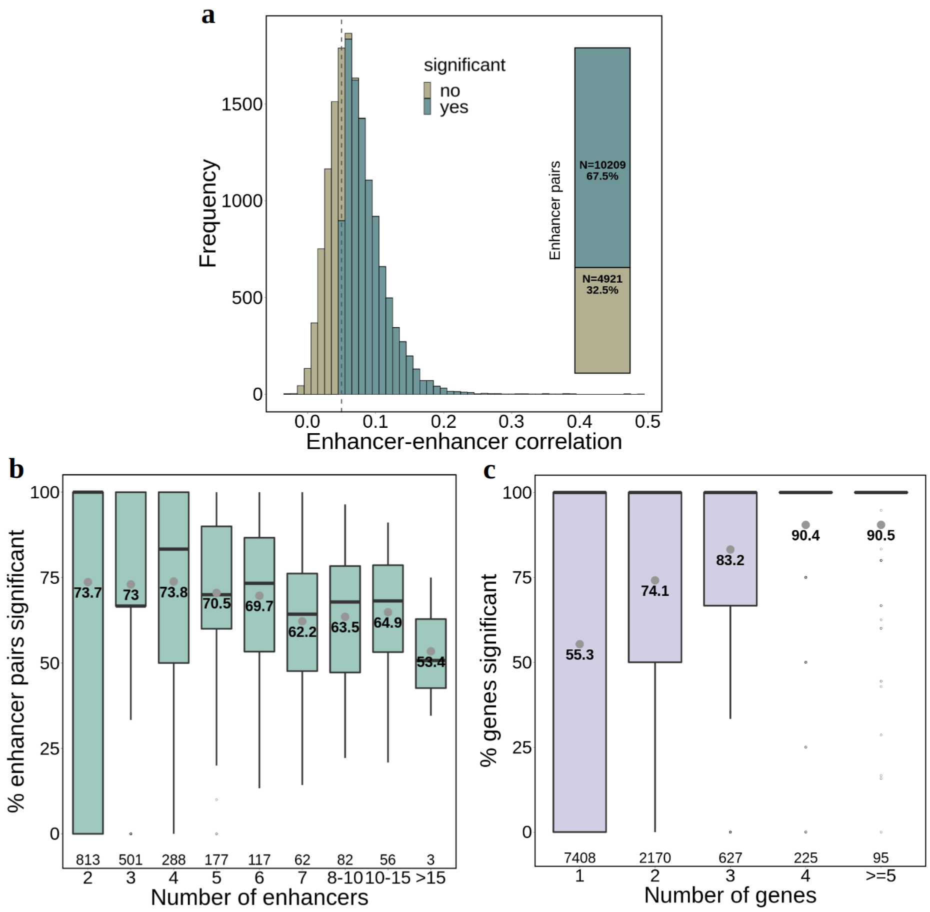

Supplementary Figure 8 Enhancer co-activity considering a 200Kb window around gene TSS. a enhancer-enhancer association correlation distribution (N = 15,130). The inner plot denotes the percentage of significant associations (green colour, FDR<5% and absolute correlation > 0.05); **b** percentage of significantly associated enhancer-enhancer pairs (y-axis) per number of enhancers significantly associated with the gene (x-axis); **c** percentage of genes in which enhancer-enhancer pairs are significantly associated (y-axis) per number of genes in which they were tested (x-axis). Note: we reduced the number of x-axis categories compared to 1 Mb results to accommodate for lower sample sizes when considering a 200Kb window.

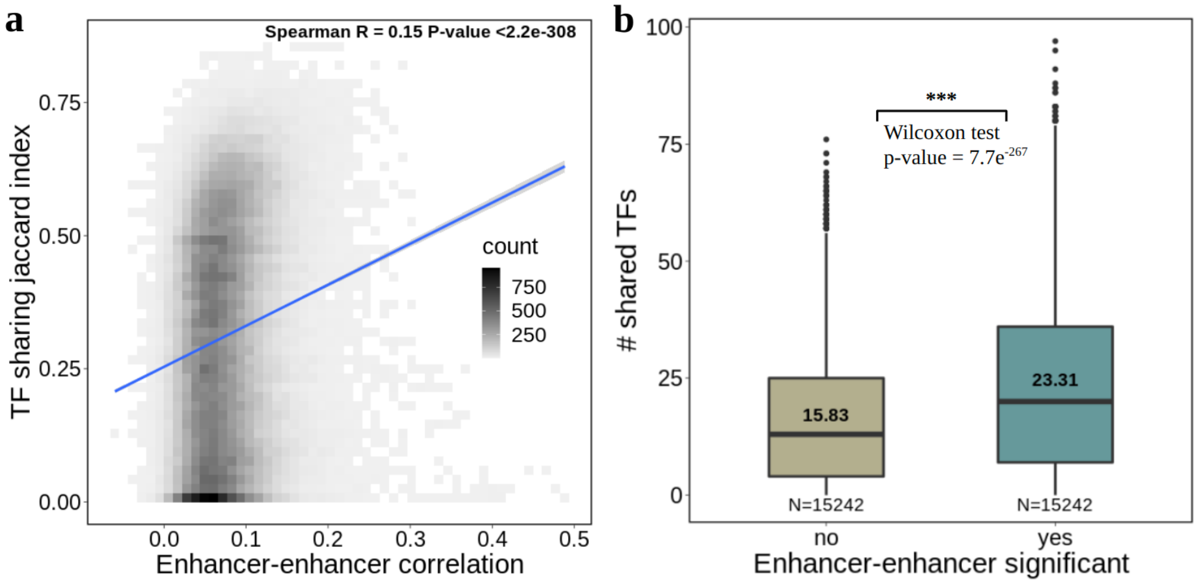

Supplementary Figure 9 Jaccard similarity index and distance-matched TF sharing using ReMap data. a TF sharing Jaccard Index (intersect of TFs found between the enhancer pair, divided by the union of TFs) per enhancer-enhancer correlation coefficient (N = 126,830). Fit line corresponds to a linear regression model with 95% confidence intervals; **b** number of distinct TFs shared between enhancer pairs depending on their association significance. Significant and non-significant enhancer pairs are a subset of all enhancer pairs that are matched for distance (see Methods). The length of the box corresponds to the IQR with the centre line corresponding to the median, the upper and lower whiskers represent the largest or lowest value no further than 1.5 × IQR from the third and first quartile, respectively. Values above the median line represent the mean.

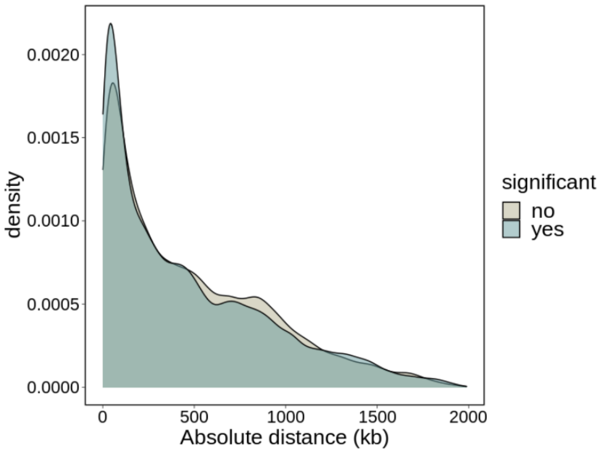

Supplementary Figure 10 Absolute distance distribution of significant and non-significant enhancer-enhancer associations. The midpoint position of the enhancer start and end coordinates was used to calculate the distance. N = 89,885 for significant enhancer-enhancer pairs and N = 36,945 for non-significant enhancer-enhancer pairs.

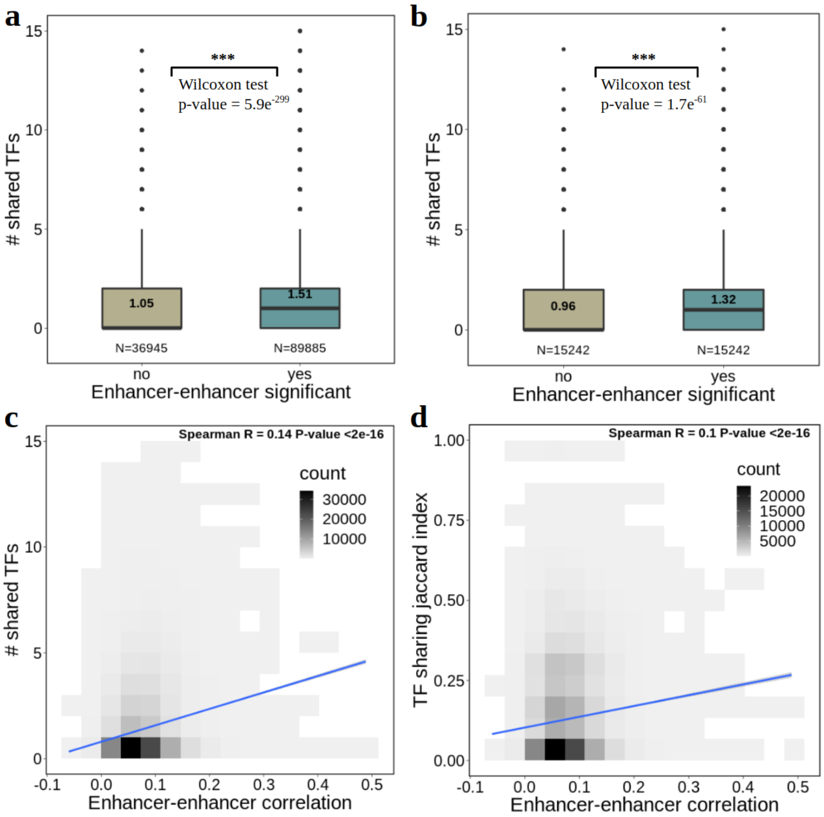

Supplementary Figure 11 Transcription factor sharing using MotifMap data. a number of distinct TFs with binding sites in both enhancers of an enhancer-enhancer pair (shared TFs), depending on their association significance. The length of the box corresponds to the IQR with the centre line corresponding to the median, the upper and lower whiskers represent the largest or lowest value no further than 1.5 × IQR from the third and first quartile, respectively. Values above the median line represent the mean; **b** same as previous, but significant and non-significant enhancer pairs are matched for distance (see Methods); **c** number of shared TFs per enhancer-enhancer correlation coefficient (N = 18,688). Fit line corresponds to a linear regression model with 95% confidence intervals; **d** TF sharing Jaccard Index (intersect of TFs between the enhancer pair, divided by the union of TFs) per enhancer-enhancer correlation coefficient (N = 18,688). Fit line corresponds to a linear regression model with 95% confidence intervals.

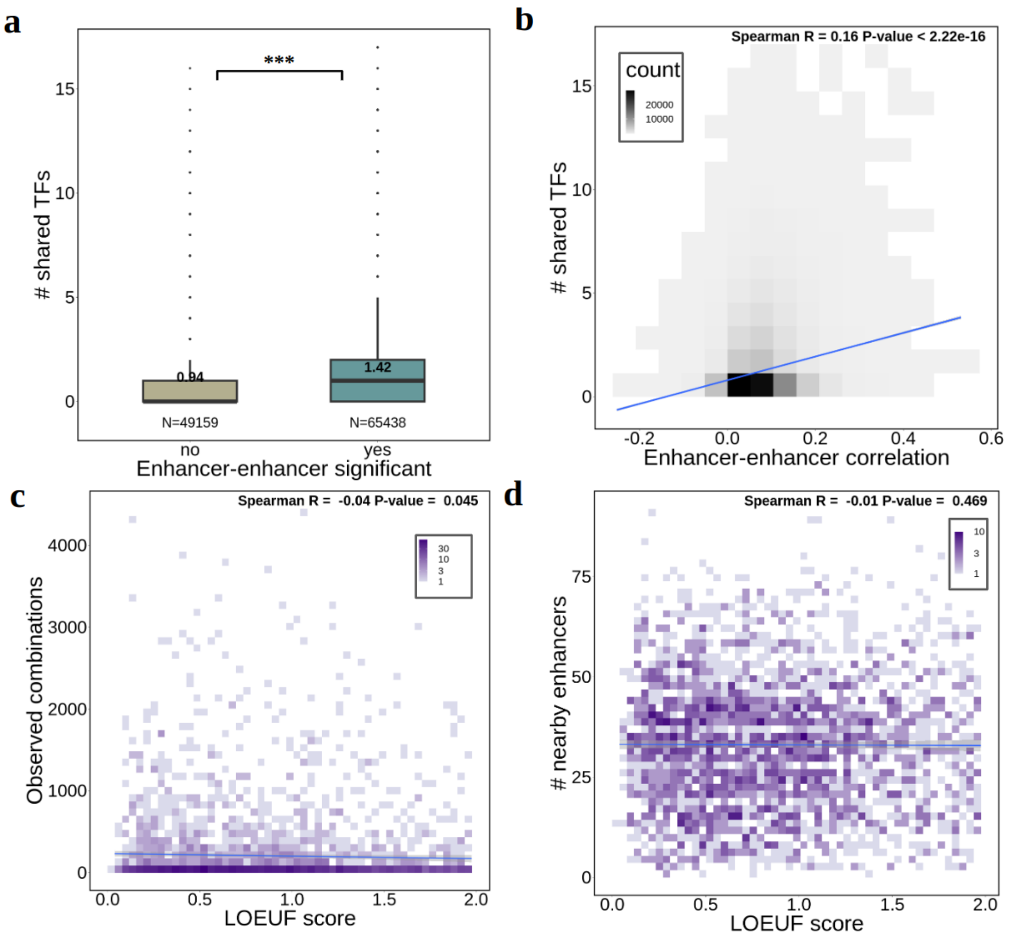

Supplementary Figure 12 Features of enhancer co-activity in the PBMC multimodal dataset. a number of distinct TFs with binding sites (MotifMap data) in both enhancers of an enhancer-enhancer pair (shared TFs), depending on their association significance. "***" denotes two-tailed Wilcoxon test p-value < $2.2e^{-16}$. The length of the box corresponds to the IQR with the centre line corresponding to the median, the upper and lower whiskers represent the largest or lowest value no further than 1.5 × IQR from the third and first quartile, respectively. Values above the median line represent the mean; **b** number of shared TFs per enhancer-enhancer correlation coefficient (N = 114,597); **c** number of enhancer combinations observed in at least one cell (y-axis) per gene LOEUF score (x-axis) (N = 2790); **d** number of enhancers within 1Mb of the gene TSS (regardless of gene-enhancer association significance) per gene LOEUF score (N = 2790). Fit lines represent a linear regression model.

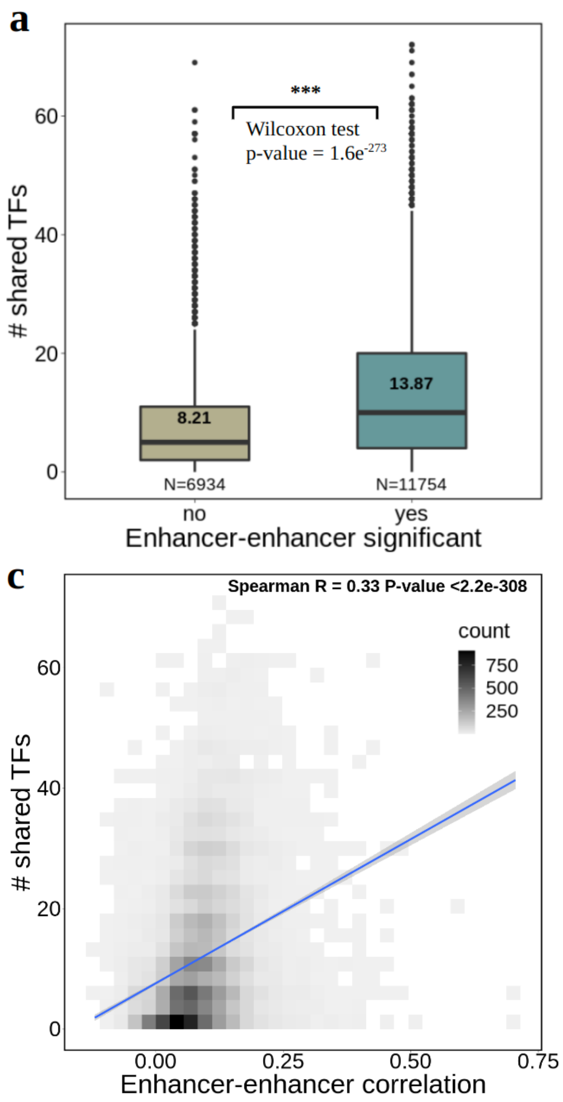

Supplementary Figure 13 Transcription factor sharing on ABC enhancers using ReMap data. a Number of distinct TFs with binding sites in both enhancers of an enhancer-enhancer pair (shared TFs), depending on their association significance. The length of the box corresponds to the IQR with the centre line corresponding to the median, the upper and lower whiskers represent the largest or lowest value no further than 1.5 × IQR from the third and first quartile, respectively. Values above the median line represent the mean; **b** Same as previous, but significant and non-significant enhancer pairs are matched for distance (see Methods); **c** Number of shared TFs per enhancer-enhancer correlation coefficient (N = 18,688). Fit line corresponds to a linear regression model with 95% confidence intervals; **d** TF sharing Jaccard Index (intersect of TFs between the enhancer pair, divided by the union of TFs) per enhancer-enhancer correlation coefficient (N = 18,688). Fit line corresponds to a linear regression model with 95% confidence intervals.

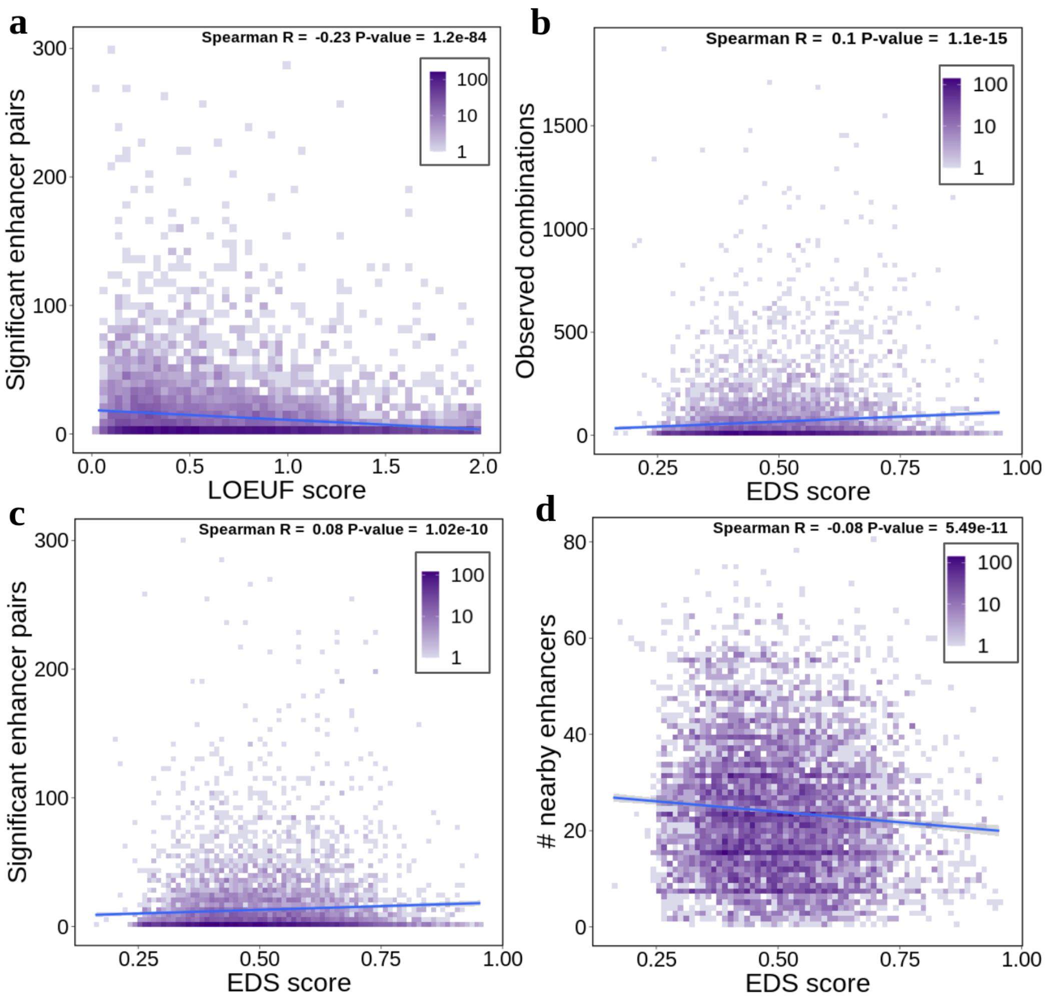

Supplementary Figure 14 Enhancer correlation comparison with gene essentiality and enhancer redundancy scores. a number of significant enhancer-enhancer pairs and gene LOEUF score (N = 6895); **b** number of enhancer combinations observed in at least one cell per enhancer-domain score (EDS, N = 6925); **c** number of significant enhancer-enhancer pairs and EDS score; **d** number of enhancers within 1Mb of the gene TSS (regardless of gene-enhancer association significance) per EDS score. Fit lines correspond to a linear regression model.

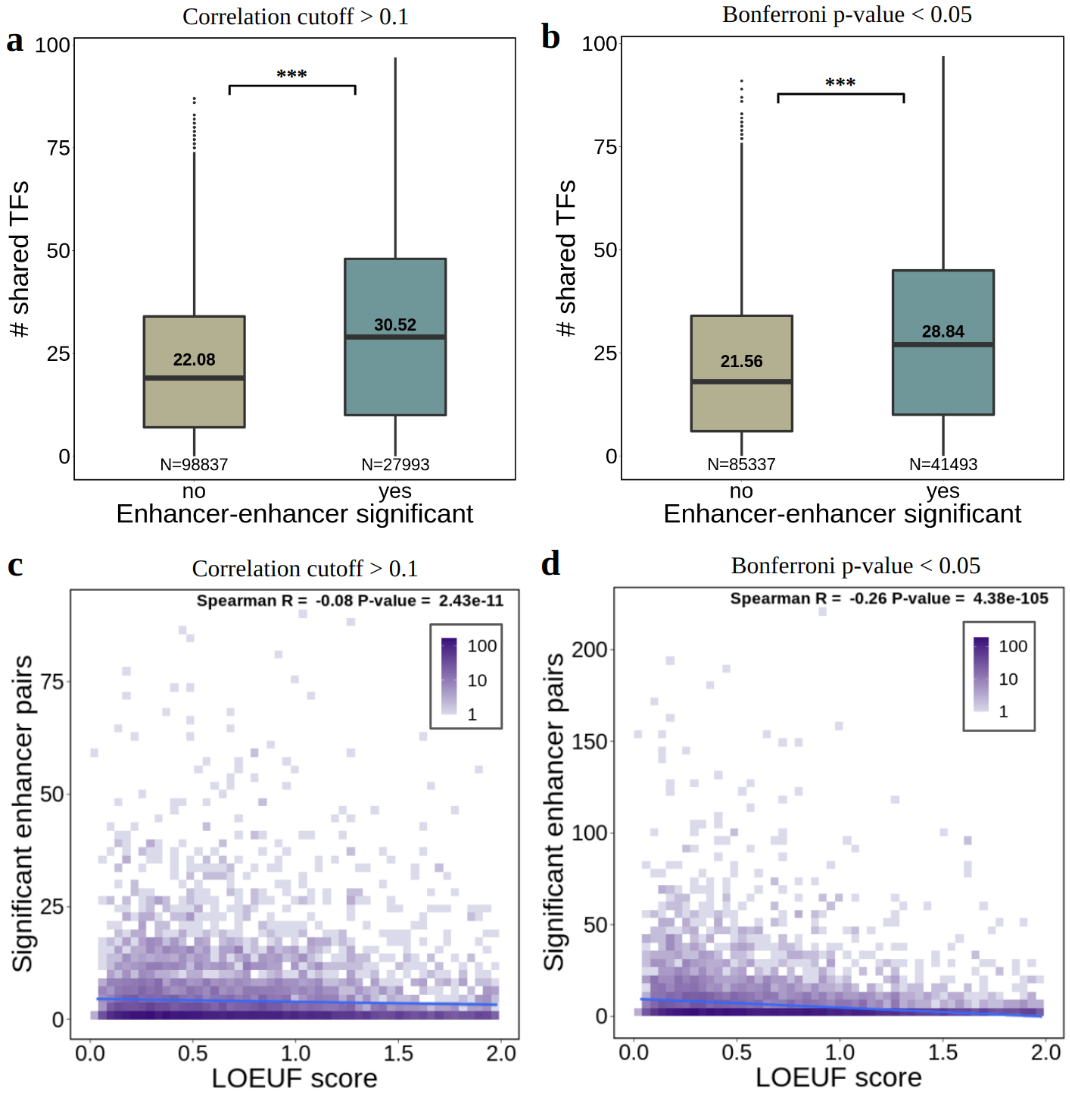

Supplementary Figure 15 Enhancer co-activity features with alternative correlation cutoffs. a number of distinct TFs with binding sites (ReMap data) in both enhancers of an enhancer-enhancer pair (shared TFs), depending on significance based on correlation > 0.1 and FDR < 5%. "***" denotes two-tailed Wilcoxon test p-value < $2.2e^{-16}$. The length of the box corresponds to the IQR with the centre line corresponding to the median, the upper and lower whiskers represent the largest or lowest value no further than 1.5 × IQR from the third and first quartile, respectively. Values above the median line represent the mean **b** same as previous but significance defined as Bonferroni-corrected p-value < 0.05; **c** gene LOEUF score (x-axis) per number of enhancer-enhancer associations with correlation > 0.1 and FDR < 5%; **d** same as previous, but for enhancer-enhancer associations with Bonferroni-corrected p-value < 0.05.

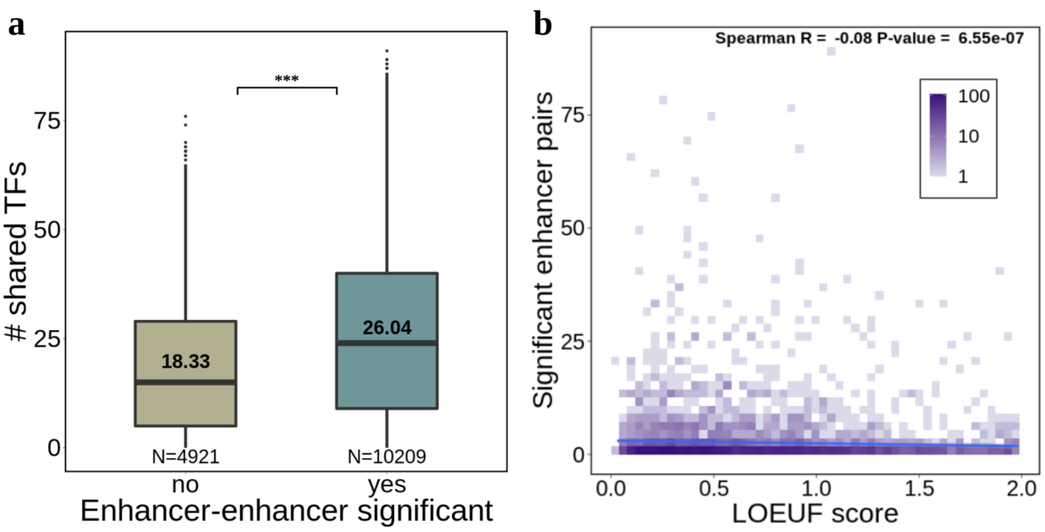

Supplementary Figure 16 Features of enhancer co-activity considering a 200Kb window around gene TSS. a number of distinct TFs with binding sites (ReMap data) in both enhancers of an enhancer-enhancer pair (shared TFs), depending on their association significance. "***" denotes two-tailed Wilcoxon test p-value < $2.2e^{-16}$. The length of the box corresponds to the IQR with the centre line corresponding to the median, the upper and lower whiskers represent the largest or lowest value no further than 1.5 × IQR from the third and first quartile, respectively; **b** number of significant enhancer-enhancer pairs and gene LOEUF score.
