# Supplementary material for: Multimodal single cell analysis infers widespread enhancer co-activity in a lymphoblastoid cell line
Source: Commun Biol. 2023 May 26;6:563. doi: 10.1038/s42003-023-04954-4 (PMC10219981; doi:10.1038/s42003-023-04954-4)
Supplement: Supplementary file 2 — Description of Additional Supplementary Files [file 42003_2023_4954_MOESM2_ESM.docx]

**Description of Additional Supplementary Files**

**File name:** Supplementary Data 1

**Description:** List of 126830 enhancer-enhancer associations tested, between enhancers significantly associated with genes.

**File name:** Supplementary Data 2

**Description:** List of 2878013 enhancer-enhancer associations between enhancers up to 1MB from gene.

**File name:** Supplementary Data 3

**Description:** List of 126830 enhancer-enhancer associations tested with partial correlation accounting for gene expression.

**File name:** Supplementary Data 4

**Description:** List of associations between enhancers chr6_26104800_26105400 and chr6_26189200_26191000.

**File name:** Supplementary Data 5

**Description:** Lists of significant enhancer-enhancer associations for different correlation and p-value cutoffs (3 lists separated by excel tabs).

**File name:** Supplementary Data 6

**Description:** Source data for main figures.
